# Radiological characteristics of coal-to-ash transformation: a paired coal-ash assessment of natural radionuclides

**DOI:** 10.1007/s10653-026-03509-w

**Published:** 2026-09-29

**Authors:** Aneta Łukaszek-Chmielewska, Marzena Rachwał, Joanna Rakowska, Barbara Piotrowska, Krzysztof Isajenko, Mirosław Szyłak-Szydłowski

**Affiliations:** 1 Fire University, Słowackiego Street 52/54, 01-629 Warsaw, Poland; 2 Central Laboratory for Radiological Protection, Konwaliowa Street 7, 03-194 Warsaw, Poland; 3 Faculty of Environmental Engineering, Warsaw University of Technology, Nowowiejska Street 20, 00-653 Warsaw, Poland

**Keywords:** Hard coal, Bottom ash, NORM, Radionuclide enrichment, Radiological transformation, Environmental exposure

## Abstract

Naturally occurring radioactive materials (NORM) in coal and its combustion residues represent a potential source of environmental contamination, particularly under large-scale energy production and waste reuse scenarios. However, the radiological transformation of radionuclides during coal combustion remains insufficiently quantified. This study applies a paired coal–ash approach to assess systematic changes in the activity concentrations of ^40^K, ^226^Ra, and ^232^Th and associated radiological hazard indices. Nine hard coal samples from Poland and international sources, together with their corresponding bottom ashes produced under controlled combustion conditions, were analysed using gamma-ray spectrometry. All coal samples exhibited low radionuclide activities, with concentrations below global average values. In contrast, combustion resulted in a highly consistent and approximately six-fold increase in radionuclide activity and all radiological indices, accompanied by complete directional consistency and very large effect sizes (r = 0.889). This increase reflects the concentration of naturally occurring radionuclides within the mineral residue, as the organic fraction of coal is removed during combustion. These findings demonstrate that combustion acts as a predictable process of radionuclide enrichment and radiological amplification. Enrichment factors ranged from approximately 3.8 to 9.9 across the analysed coal–ash pairs, and the external gamma radiation dose rate exceeded the recommended reference level of 54 nGy h⁻^1^ in all nine ash samples. Although most ash samples remained within recommended radiological limits, the magnitude and uniformity of enrichment indicate a potential risk of secondary environmental contamination and increased environmental exposure—particularly where combustion residues are reused in construction materials or land applications. It should be noted, however, that this study assessed the potential for such impact based on elevated radioactive activity of the ash, rather than on direct observation of radionuclide migration into the environment. The results highlight the need to treat coal combustion residues as radiologically distinct materials—at least under the combustion conditions investigated in the present study—and to incorporate transformation processes into environmental risk assessment and management frameworks.

## Introduction

Hard coal is a crucial raw material for ensuring global energy security. It is classified as a primary, non-renewable energy source. Poland has significant reserves of this resource, estimated at approximately 64.6 billion tons (Szuflicki et al., 2025) mined primarily in the Upper Silesian Coal Basin (USC) and the Lublin Coal Basin (LZW). In 2022, nearly 70% of Poland’s electricity production came from coal, but in 2024, this figure dropped to 57%. Hard coal production in 2024 decreased by 8.9% compared to 2023, reaching 44.22 million tons (Szuflicki et al., 2025). In 2022, Poland imported 16.7 million tons of hard coal, including 5.8 million tons for the commercial and industrial power sectors. The main reason for this import in 1990 and 2022 was the growing cost of mining in Poland (Miniszewski & Pilszyk, 2023). In this context, the presence and behaviour of naturally occurring radioactive materials in coal and its combustion products represent an important, yet often underappreciated, aspect of environmental impact.

Coal combustion residues are widely recognised as technologically enhanced naturally occurring radioactive materials (TENORM), which may act as secondary sources of environmental contamination. Large volumes of coal ash are commonly reused in construction materials, road base layers, and land applications, creating potential pathways for radionuclide dispersion into soil, groundwater, and the atmosphere. In such scenarios, the radiological impact is not limited to the combustion process itself but extends to long-term environmental exposure and material recycling systems. Therefore, understanding the transformation of radionuclide concentrations during combustion is essential for assessing the full environmental footprint of coal use.

Hard coal is a sedimentary rock of organic origin formed through biological, biochemical, and geochemical transformations of accumulated organic matter (Starzycka et al., 2020). It consists of organic material, mineral phases, and trace elements, including naturally occurring radioactive isotopes such as ^238^U, ^226^Ra, ^232^Th, and ^40^K, whose concentrations depend on mineral composition and coal properties (Adamczyk et al., 2023; Olkuski & Stala-Szlugaj, 2009; Róg, 2005). The radionuclide content varies with grain size and maceral composition, with higher concentrations often associated with fine fractions and mineral-rich components (Róg, 2005; Wysocka et al., 2019)(Olkuski & Stala-Szlugaj, 2009). In coal deposits generally, uranium is commonly associated with mineral phases such as pyrite- and calcite-rich sandstones, while accessory minerals including phosphates, zircon, monazite, xenotime, and uranothorite are recognised as key carriers of radioactivity (Nieć, 2009). The presence of these specific mineral phases was not directly verified in the coal samples analysed in the present study, as no mineralogical characterisation (e.g., X-ray diffraction or scanning electron microscopy) was performed; the radionuclide behaviour discussed here is inferred from gamma-ray spectrometric activity concentrations rather than from direct mineralogical evidence. This mineralogical diversity implies that the behaviour of radionuclides during combustion—and consequently their distribution between gaseous and solid combustion products—may vary significantly depending on the mineralogical composition of the parent coal (Ahmed & Joubert, 2019; Aloraini & El-Azony, 2020; Celenlioglu & Karali, 2025; Parzentny & Róg, 2019).

The behaviour of radionuclides during coal combustion is a complex, process-dependent phenomenon that has been the subject of increasing scientific attention. As the organic fraction of coal is oxidised during combustion, radionuclides associated with mineral phases become concentrated in the remaining solid residue—fly ash and bottom ash—leading to activity concentrations typically 3 to 10 times higher than those in the parent coal (Bhangare et al., 2014; Deonarine et al., 2023). However, the magnitude of this enrichment is not uniform: it depends on the mineralogical form in which radionuclides occur in coal, the combustion temperature and technology, residence time, and the type of ash fraction collected (Krylov & Sidorova, 2013). For instance, ^40^K, which is partly associated with volatile mineral forms, tends to show lower and more variable enrichment in bottom ash compared to ^226^Ra and ^232^Th, which are preferentially retained in refractory mineral phases (Bhangare et al., 2014; Walencik-Łata & Smołka-Danielowska, 2020). The combustion technology itself—whether pulverised coal, fluidised-bed, or fixed-bed grate firing—significantly affects radionuclide partitioning between fly ash and bottom ash fractions (Altıkulaç et al., 2022; Walencik-Łata & Smołka-Danielowska, 2020). Despite these well-documented dependencies, the vast majority of existing studies treat coal and ash as independent sample sets, without establishing a direct quantitative link between the radionuclide composition of a specific coal and the radiological properties of its combustion products. This gap limits the ability to assess whether combustion systematically amplifies radiological risks and to develop process-based predictive frameworks for the management of TENORM-containing residues.

Since hard coal is imported to Poland from various parts of the world, and the Earth’s crust contains heterogeneous concentrations of natural radioactive isotopes, this study pursues three specific objectives: (1) to determine the activity concentrations of ^226^Ra, ^232^Th, and ^40^K in Polish and imported hard coals representing diverse geological origins; (2) to quantify the magnitude and consistency of radionuclide enrichment and radiological index amplification resulting from combustion, using a paired coal–ash design in which each ash sample is directly linked to its parent coal; and (3) to assess the environmental implications of the observed transformation in the context of coal combustion residue management and reuse. During hard coal combustion, only the organic matter is oxidised to gaseous form, while the vast majority of contaminants—including a significant portion of natural radioactive isotopes—remain in solid combustion products, resulting in radionuclide activity concentrations approximately 7 to 10 times higher in coal combustion residuals than in the parent coal (Lauer et al., 2015), consistent with the general enrichment pattern also reported by Bhangare et al. (2014). Despite extensive research on radionuclide concentrations in coal and combustion by-products, existing studies typically evaluate coal and ash as independent materials, overlooking the combustion-driven transformation that fundamentally alters their radiological properties and environmental behaviour. The extent to which combustion systematically amplifies radiological risks in materials subsequently released into the environment or reused within circular economy systems, therefore, remains insufficiently quantified. In this context, the present study applies a paired coal-ash approach to quantify the magnitude and consistency of radiological transformation during combustion, providing a process-based perspective on the environmental implications of coal-derived waste. We hypothesise that combustion acts as a systematic and predictable amplifier of radionuclide activity, and that the resulting enrichment is sufficiently consistent across coal types to be quantified and incorporated into environmental risk assessment frameworks.

## Methodology

### Sample collection and preparation

Nine samples of hard coal were collected from shipments transhipped at the Port of Gdańsk using a random representative sampling procedure in accordance with ISO 18283:2008. Each sample was collected from a different shipment, representing coal from a distinct geographical origin, and was therefore treated as an independent observation. The Australian samples (C1, C6) originated from the Bowen Basin, Queensland—a Permian–Triassic bituminous coking coal basin—comprising one sample from the Hail Creek mine and one corresponding to the Premium Low-Volatile (PLV) export trade grade. The Polish sample labelled as Upper Silesian Coal (C8) originated from the Carboniferous Upper Silesian Coal Basin, while the second Polish sample (‘Poland Mix’, C2) is presumed to derive predominantly from the same basin. The US samples (C4, C7) originated from the Central Appalachian coalfield, Virginia—a Pennsylvanian bituminous coal region—with C7 specifically sourced near Clintwood, Virginia. The Kazakh sample (C3) is associated with the Carboniferous Karaganda–Ekibastuz coal basins. The Mozambican sample (C5) originated from the Permian Moatize Basin (Karoo Supergroup), and the Colombian sample (C9) from the Paleogene Cerrejón Formation. Overall, the analysed coals span depositional ages from the Carboniferous to the Paleogene across markedly different tectonic and sedimentary settings, providing a geologically heterogeneous sample set. It should be acknowledged that, with one sample per source, the design does not permit formal statistical separation of source-specific effects from sample-specific variability. However, the consistency of the observed combustion-induced enrichment across this geologically heterogeneous sample set provides evidence that the amplification effect is not confined to a single coal type or geological origin, even though formal replication within each source would be required to statistically isolate source-specific effects from sample-specific variability.

**Fig. 1:**
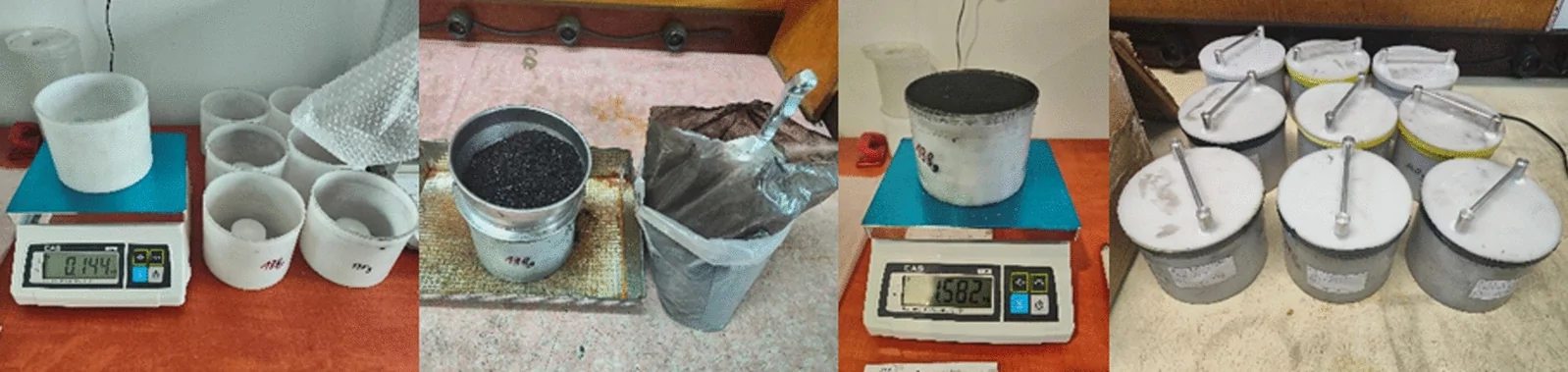
The process of preparing samples for testing (from left to right): weighing of empty Marinelli vessels prior to filling; filling of a Marinelli vessel with a crushed and ground coal sample; weighing of a filled Marinelli vessel to determine the net sample mass; sealed Marinelli vessels prepared for gamma-ray spectrometric measurement

To perform radioactivity tests, coal samples were mechanically crushed and ground in a porcelain mortar. The samples were then sieved through 2-mm mesh sieves and placed in cylindrical Marinelli vessels with a capacity of 1.7 L. This crushing and sieving procedure was applied specifically to standardise particle size across all samples and to minimise the potential influence of coal particle or lump size on measurement homogeneity, representativeness, and repeatability. Prior to processing, the samples varied in lump size as received (ranging from fine fractions to coarse lumps depending on the shipment, e.g., 2–13.5 mm for one certified shipment and 10–50 mm for another); reducing all samples to a uniform sub-2-mm fraction ensured comparable sample homogeneity across the diverse coal sources analysed. The containers were filled with samples up to 5 mm from the top edge, hermetically sealed and stored for four weeks until radioactive equilibrium was reached between ^226^Ra and ^214^Bi, and ^232^Th and ^208^Tl. The net mass of each sample was then determined. The concentrations of ^232^Th and ^226^Ra were determined indirectly—a method commonly applied in gamma-ray spectrometry—by measuring the activity of their secular equilibrium daughter products: ^208^Tl for ^232^Th and ^214^Bi for ^226^Ra, respectively. Measurements were performed nine times for each sample without removing or repositioning it between consecutive measurements, thereby ensuring a consistent measurement geometry throughout. These nine repeated measurements constitute analytical replicates of a single prepared sample—performed under identical measurement geometry without repositioning—and therefore provide information on analytical (counting) repeatability rather than on the internal heterogeneity of the coal material itself. No independently prepared subsamples (i.e., separate portions of the same coal shipment, independently crushed, sieved, and packed) were analysed in this study. Consequently, the reported measurement uncertainty reflects instrumental and counting-statistics variability, and does not capture potential compositional heterogeneity within a given coal sampleThe obtained results represent the arithmetic mean of these nine measurements. Each sample was measured for 18,000 s. Figure 1 shows the sample preparation process for testing.

### Combustion process

The combustion of the hard coal samples was carried out in a Kołton solid-fuel heating boiler. Technical data and the boiler model are presented in Table 1 (data from the boiler rating plate).

**Table 1:** Technical data of the furnace

Technical data (rating plate)	
Furnace type	UNIX 20
Manufacturing year	2016
Serial number	13,517–2016-0 T-0Q
Primary fuel	Hard coal; size walnut I/II
Substitute fuel	Firewood (max. moisture content 20%)
Rated power	Coal—23 kW
Boiler class	Class 2
Efficiency	78–85%
Max. operating pressure	2.0 bar
Max. water temeperature	95 °C
Boiler water capacity	100 l
Boiler weight	310 kg
Electrical supply	230 V; 50 Hz

The coal sample combustion station was equipped with an additional 400 ml butane cartridge containing 227 g of liquefied gas. This cartridge was manufactured by Tiross (model TS-700) and was used to ignite the coal samples. The cartridge was fitted with a gas burner with a knob for regulating the flame. The coal sample was ignited using a piezoelectric igniter. Each coal sample was burned until the combustion process was complete. The boiler was then allowed to cool naturally before the resulting ash was carefully swept from the bottom of the boiler. The furnace was thoroughly cleaned before burning the next sample. The amount of coal burned in a single experiment corresponded to the sample mass placed in the Marinelli vessel, as determined prior to combustion.

The Kołton UNIX 20 is a bottom-feed, gravity-draft solid-fuel boiler operating under natural draught conditions, without forced air supply. Combustion in this type of boiler proceeds in a fixed-bed grate configuration, with combustion temperatures in the active combustion zone typically ranging from 700 to 1050 °C for hard coal, consistent with values reported for grate-fired domestic boilers. The combustion temperature was not directly measured during the experiments; the boiler was operated under standard conditions in accordance with the manufacturer’s specifications.

Although domestic grate-fired boilers differ from industrial power boilers in terms of scale, combustion temperature, and the absence of flue gas treatment systems, the fundamental mechanism of radionuclide behaviour during combustion remains analogous: the organic fraction of coal is oxidised to gaseous form, while radionuclides are retained and concentrated in the solid residue. The results obtained in this study may therefore be considered indicative of the direction and magnitude of radiological transformation during coal combustion more broadly, while acknowledging that the specific enrichment factors may vary under industrial combustion conditions."

### Experimental setup

**Fig. 2:**
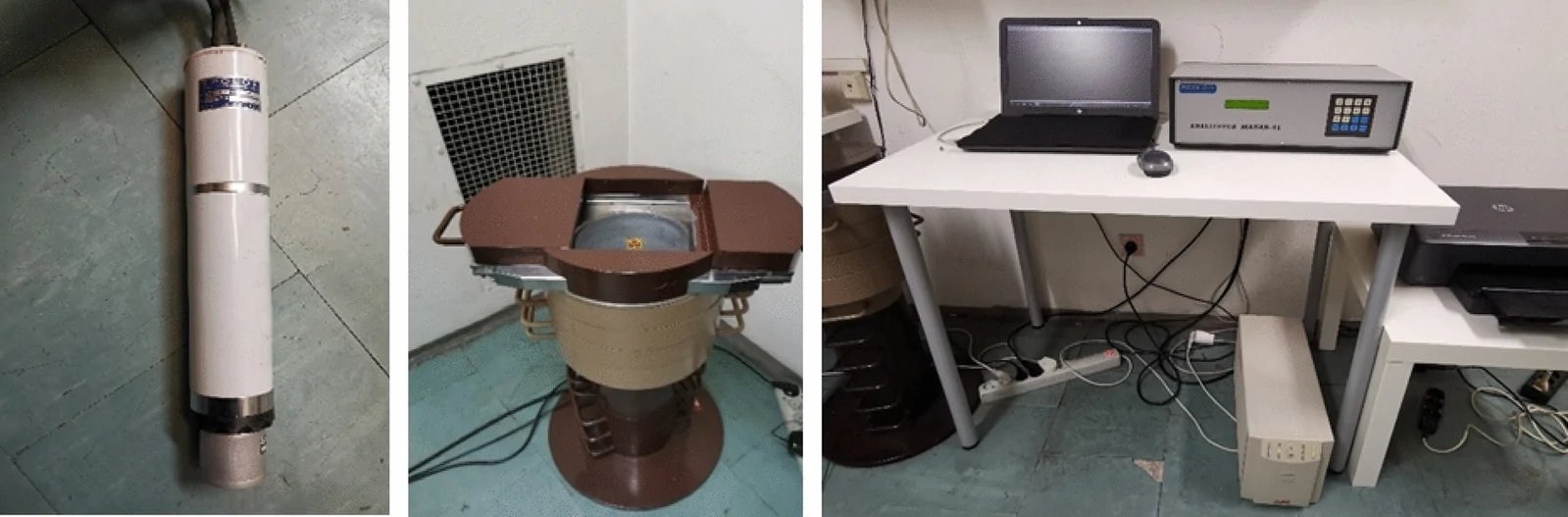
MAZAR Spectrometric Kit (from left to right): NaI(Tl) scintillation probe; probe positioned inside the lead shielding chamber with a Marinelli vessel containing the sample; complete measurement workstation comprising the MAZAR-22 three-channel scaler, laptop with dedicated software, and uninterruptible power supply (UPS)

Samples of the hard coal and ash were analysed using a MAZAR spectrometer connected to a 2 × 2" NaI(Tl) scintillation probe. This analyser operates in three measurement ranges for the determination of the concentrations of the radioactive isotopes ^226^Ra, ^232^Th and ^40^ K. The ^226^Ra and ^232^Th activity concentrations were determined by the photo peaks of their daughters, ^214^Bi (1764 keV) and ^208^Tl (2610 keV), respectively. The ^40^ K activity concentration was determined directly from the 1460 keV photopeak. The spectrometric equipment to measure the concentrations of natural radioactive isotopes in the tested samples consists of a scintillation probe, a lead shielding house, a three-channel pulse amplitude analyser, with a processor, control system, and printer, as well as a set of calibration standards (Fig. 2). The measurement system used for spectrometric measurements was the scintillation probe, which was placed in a lead shielding box to reduce the impact of background radiation on the measurement results. Electrical pulses generated in the probe by gamma radiation photons were amplified and then transmitted via single-channel analysers to a 3-channel scaler, which counted and recorded them. Calibration of the detector performance was based on measurements of three volumetric calibration standards: ^226^Ra, ^232^Th, and ^40^ K, as well as a measurement of the standard matrix as a background measurement for the calibration process. Background measurements for the tested samples were made with an aluminium equivalent of the average sample mass. The geometry of the reference sources consisted of Marinelli-type containers with a capacity of 1.7 dm^3^. The method also takes into account the self-absorption of gamma radiation in the studied sample material. The energy resolution of the spectrometer was 6–8% (Drzymała et al., 2020). Sand was used as the reference material. Only certified reference materials from the U.S. Department of Energy New Brunswick Laboratory were used to produce Ra-226 and Th-232 standards. The potassium standard was made using chemically pure potassium chloride. The matrix was sand with a density of 1.6 kg/dm^3^. The reliability of the measurements was assessed based on the combined relative measurement uncertainty, which was typically within 10–15% for ^226^Ra and ^232^Th and within 10–12% for ^40^K, arising from counting statistics, detector efficiency calibration, and background variability (Isajenko et al., 2025). The minimum detectable activities (MDA) for the applied measurement geometry were on the order of a few Bq kg^−1^ for ^226^Ra and ^232^Th, and below 20 Bq kg^−1^ for ^40^K, which is sufficient for the assessment of NORM-containing materials at the activity levels encountered in this study. The self-absorption correction for gamma radiation was performed automatically by the instrument software, using the matrix-based calibration approach implemented in the MAZAR system.

### Hazards related to the presence of natural radioactive isotopes in hard coal

Hard coal, as a mineral raw material, as well as bottom ash being a waste after coal burning, contain natural radioactive isotopes that are part of the natural radioactive series and are therefore classified as a Naturally Occurring Radioactive Material (NORM). To assess human exposure to ionising radiation emitted by coal and ash, the following radiological indicators were determined: radium concentration equivalent (Ra_eq_), external exposure index (H_ex_), external gamma radiation dose rate (D_ex_), annual external effective dose (AED_ex_), and radioactivity concentration index (Iγ). These parameters represent the most widely adopted set of screening indices in international radiological protection frameworks and in the scientific literature on NORM-containing materials (UNSCEAR, 2000; EC RP 112). Ra^eq^ provides a unified measure of the combined gamma radiation hazard from ^226^Ra, ^232^Th, and ^40^K. H_ex_ allows direct comparison with the commonly applied safety threshold of 370 Bq kg^−1^. D_ex_ and AED_ex_ quantify the actual external gamma dose received, enabling comparison with reference dose levels for the general public; and Iγ serves as a regulatory screening index for building materials under the Euratom Basic Safety Standards. A detailed description of the calculated radiological parameters can be found in the publications (Drzymała et al., 2020; Lauer et al., 2015; Pak et al., 2025).

### Statistical analysis

Normality of distributions was assessed using the Shapiro–Wilk test for the activity concentrations of ^40^K, ^226^Ra, and ^232^Th. These tests were performed on the pooled dataset of all analysed coal samples (n = 47), combining the nine coal samples of the present study with thirty-eight literature-derived samples (Table 3), rather than separately for each subgroup (present-work vs. literature-derived, or coal vs. ash). As all three radionuclides significantly deviated from normality (*p* < 0.05; see Sect. “Statistical analysis” for detailed test statistics), non-parametric methods were applied throughout the subsequent analysis, including Spearman’s rank correlation, the Friedman test, and Wilcoxon signed-rank tests. Outliers were identified using the interquartile range (IQR) method (Q1 − 1.5 × IQR; Q3 + 1.5 × IQR), applied to the same pooled dataset for both the natural radionuclides (^40^K, ^226^Ra, ^232^Th) and the derived radiological hazard indices (Raeq, Hex, D, AED, and I).

Monotonic relationships between radionuclides were evaluated using Spearman’s rank correlation coefficient (ρ). Outliers were identified using the interquartile range (IQR) method (Q1 − 1.5 × IQR; Q3 + 1.5 × IQR).

Differences in activity concentrations of ^40^K, ^226^Ra and ^232^Th were tested using the Friedman test for related samples, followed by post‑hoc pairwise comparisons using the Wilcoxon signed‑rank test with Bonferroni correction and the Nemenyi test.

A paired coal–ash analysis was used to quantify combustion‑induced changes in radionuclide activity and radiological indices. The Wilcoxon signed‑rank test (alternative: ash > coal) and effect size (r) were calculated, and ash‑to‑coal ratios were used to characterise the magnitude of transformation.

All analyses were conducted in R version 4.4.2, using the following packages: readxl, tidyr, dplyr, ggplot2, cowplot, and PMCMRplus.

## Results and discussion

### Concentrations of the natural radioactive isotopes in hard coal

The concentrations of the natural radioactive isotopes in the hard coal samples varied within a relatively narrow range (Table 2). The lowest concentrations of the natural radioactive isotopes ^226^Ra, ^232^Th, and ^40^ K were obtained for the C9 sample from Colombia, while the highest concentrations of ^226^Ra and ^232^Th were found in the C5 sample from Mozambique. The Polish hard coal sample, C8, contained the highest concentration of ^40^ K. For none of the coal samples tested was there a significant increase in the concentrations of natural isotopes of radium, thorium, and potassium compared to the worldwide average values for these concentrations in hard coal or the average content of these radionuclides in the Earth’s crust. These values are broadly consistent with previously reported activity concentrations in hard coals worldwide (Lauer et al., 2015; Lu et al., 2012); a detailed, sample-by-sample comparison against an extended literature dataset (Table 3, C10–C47) is presented below. This confirms that, despite differences in geological origin, the analysed coals do not exhibit anomalously elevated natural radioactivity.

**Table 2:** Concentration of natural radioactive isotopes ^226^Ra, ^232^Th and ^40^ K in the nine hard coal samples analysed in the present study (C1–C9)

Origin country	Sample	^226^Ra (Bq·kg^−1^)	^232^Th (Bq·kg^−1^)	^40^ K (Bq·kg^−1^)
Australia (Hail Creek)	C1	15.47 ± 5.71	11.0 ± 3.4	24 ± 22
Poland (Poland Mix)	C2	15.48 ± 5.12	< MDA^*^	< MDA^*^
Kazakhstan	C3	17.03 ± 5.43	3.6 ± 2.9	24 ± 20
USA (Virginia)	C4	20.69 ± 5.74	8.4 ± 3.1	27 ± 21
Mozambique	C5	25.28 ± 6.60	19.7 ± 3.9	55 ± 25
Australia PLV	C6	12.77 ± 6.05	8.7 ± 3.6	< MDA^*^
USA (Clintwood)	C7	22.58 ± 6.36	5.6 ± 3.3	45 ± 24
Poland USC	C8	19.22 ± 5.61	11.5 ± 3.2	100 ± 25
Colombia	C9	5.66 ± 4.48	10.0 ± 3.5	50 ± 25
Average worldwide isotope concentrations in hard coals (UNSCEAR, 2000)		24	22	100
Average worldwide isotope concentrations in the Earth’s crust (UNSCEAR, 2008)		32	45	412

^*^MDA—the minimum detectable amounts or situation when the uncertainty was higher than obtained result. Although below the MDA threshold, the measured activity concentrations for these radionuclides were retained as point estimates and used throughout the subsequent calculations and statistical analyses, consistent with common practice in radiological screening; these measurements carry a correspondingly higher relative uncertainty, as reflected in the reported error margins

**Table 3:** Concentrations of natural radioactive isotopes in hard coals reported in the literature (C10–C47)

Country	Sample	Concentration, Bq·kg^−1^			References
		^238^U (^226^Ra)	^232^Th	^40^ K	
Great Britain	C10	15	7.5	144	(Smith et al., 2001)
Ireland	C11	30 (6–67)	8 (2–13)	61 (20–100)	(Organo et al., 2004)
Tanzania	C12	41.3 ± 1.0	37.2 ± 1.1	293 ± 14	(Makundi et al., 2018)
Serbia	C13	29	31	120	(Janković et al., 2011)
China	C14, C19, C22	26–40	37–37.5	59–105.7	(Lu et al., 2006, 2012)
Turkey	C15, C25, C38-C39	11.12–159.6	20–123.01	14.15–321.7	(Akkurt et al., 2009; Cevik et al., 2008; Ozmen et al., 2024)
Spain	C16	30	23	242	(Charro & Pena, 2013)
India	C17	24	39	83	(Mishra, 2004)
Brazil	C18	321	22	191	(Flues et al., 2007)
Bangladesh	C20	54.3	92.39	241	(Habib et al., 2019)
India	C21	16.8	19.5	37.2	(Sahu et al., 2014)
Nigeria	C23	8.18	6.97	27.38	(Kolo et al., 2016)
USA	C24, C44-C46	7.4–30 (8–14)	6.3–14	27	(Coles et al., 1978; Lauer et al., 2015)
Australia	C26-C34	9–47 (19–24)	11–69	23–140	(Fardy et al., 1989; IAEA, 2003)
Germany	C35	10–145	10–63	10–700	(IAEA, 2003)
Ukraine	C36	76	21	300	(Pak et al., 2021)
Russia	C37	12.3	800	780	(Pak et al., 2021)
Kazakhstan	C40-C43	20–27.9 (18.4–22)	12.0–26.2	<23–97.0	(Pak et al., 2025)
Poland	C47	18		59	(Róg, 2005)

Samples C4, C5 and C7 contained radium isotope concentrations comparable to the concentration of this radionuclide in hard coals mined worldwide. For thorium and potassium isotopes, samples from Mozambique (C5) and Poland (C8) contained comparable concentrations of ^232^Th and ^40^ K as hard coals from other places in the world. Based on this, it can be concluded that hard coals imported to Poland do not exhibit elevated radioactivity.

The differences in radionuclide concentrations observed among the analysed samples are consistent with the mineralogical diversity of the source deposits: higher ^226^Ra and ^232^Th concentrations in the Mozambique sample (C5) likely reflect a greater abundance of accessory mineral phases such as phosphates and monazite, whereas the low concentrations in the Colombian sample (C9) suggest a predominantly organic-rich, mineral-poor coal composition.

**Fig. 3:**
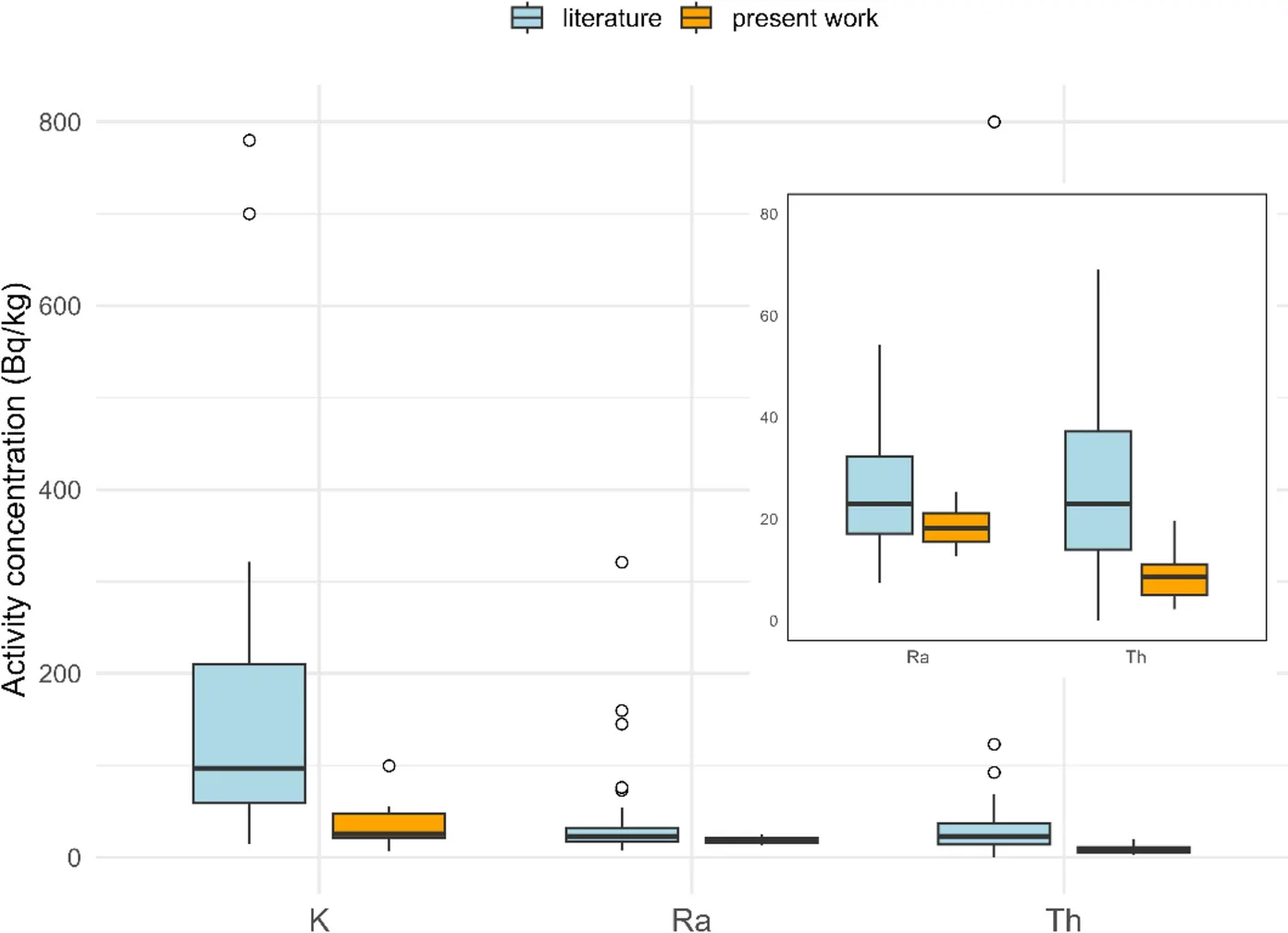
Boxplot comparison of activity concentrations (Bq·kg^−1^) of ^40^ K, ^226^Ra, and ^232^Th in coal samples from various countries, grouped by source: present study (orange) and literature data (light blue). Outliers are shown as white-centered circles. The inset in the lower right corner highlights the ^226^Ra and ^232^Th distributions without outliers and with a restricted y-axis (0–80 Bq·kg^−1^) for better comparison within the typical concentration range

The concentrations of natural radioactive isotopes for coal from various parts of the world (Table 3) indicate a wide range of these values. Average concentrations of the ^226^Ra, ^232^Th, and ^40^ K isotopes ranged from 7.4 to 321 Bq·kg^−1^, 6.3 to 800 Bq·kg^−1^, and 14.55 to 780 Bq·kg^−1^, respectively. The highest radium isotope concentration was obtained for a C18 coal sample from Brazil. The ^226^Ra concentration in this sample was over 13 times higher than the worldwide average concentration of this isotope for hard coals. For the ^232^Th and ^40^ K isotopes, the highest concentrations of these isotopes were obtained for a C37 sample from the Urtuisk deposit, Russia. The concentrations of these isotopes were approximately 36- and 8-fold higher, respectively, compared to the average concentration of these radionuclides for coals from around the world. Boxplots of K, Ra and Th concentration activity (Fig. 3) based on all samples (C1–C47) make these differences more visible. The literature data presented in Table 3 indicate substantial variability in radionuclide concentrations across different regions, with some reported values exceeding global averages by more than an order of magnitude. Compared to these datasets, the coal samples analysed in this study are located within the lower range of reported values, suggesting relatively low radiological significance of both domestic and imported coals considered here.

It should also be noted that the literature data compiled in Table 3 originate from studies performed using different analytical methods and measurement procedures, which may partially account for the observed variability among individual publications and should be considered when making direct comparisons (Cevik et al., 2008; Kolo et al., 2016; Lauer et al., 2015).

### Assessment of radiological indicators

To assess radiation exposure, the radium equivalent activity (Ra_eq_), external concentration index (H_ex_), external gamma radiation dose rate (D_out_), external annual effective dose (AED_out_), and radioactivity concentration index (I_γ_) of the tested samples were determined. A detailed description of the studied indicators is presented in several publications (Drzymała et al., 2020; Łukaszek-Chmielewska et al., 2024a, 2024b). The results of the calculations of the radium equivalent activity, external concentration index, external gamma radiation dose rate, external annual effective dose and radioactivity concentration index of the tested samples are presented in Table 4. To compare hard coals containing different concentrations of natural radioactive isotopes ^226^Ra, ^232^Th and ^40^K, the radium equivalent Ra_eq_ was calculated, which assumes that concentrations of 370 Bq·kg^−1^^226^Ra, 259 Bq·kg^−1^^232^Th and 4810 Bq·kg^−1^^40^K are the source of the same γ radiation dose in the environment (Beretka & Mathew, 1985). The maximum Ra_eq_ should be less than 370 Bq·kg^−1^ for safe use as recommended by the Organisation for Economic Cooperation and Development (NEA, 1979). The average values of the Ra_eq_ index were within the range of 19.3–57.7 Bq·kg^−1^. For none of the tested coal samples was the value of this index exceeded, i.e. 370 Bq·kg^−1^.

**Table 4:** Radiological parameter values for the tested hard coal samples

Sample ID	Ra_eq_ (Bq·kg^−1^)	H_ex_ (-)	D_ex_ (nGy·h^−1^)	AED_out_ (mSv·y^−1^)	I_γ_ (-)
C1	33.1 ± 7.7	0.089 ± 0.021	14.8 ± 3.5	0.018 ± 0.004	0.11 ± 0.03
C2	19.3 ± 6.6	0.052 ± 0.018	8.8 ± 3.0	0.011 ± 0.004	0.065 ± 0.02
C3	24.0 ± 7.0	0.065 ± 0.019	11.0 ± 3.2	0.014 ± 0.004	0.083 ± 0.02
C4	34. 8 ± 7.5	0.094 ± 0.020	15.8 ± 3.4	0.019 ± 0.004	0.12 ± 0.03
C5	57.7 ± 8.9	0.156 ± 0.024	25.9 ± 4.0	0.032 ± 0.005	0.20 ± 0.03
C6	26.2 ± 8.1	0.071 ± 0.022	11.7 ± 3.7	0.014 ± 0.004	0.09 ± 0.03
C7	34.1 ± 8.2	0.092 ± 0.022	15.7 ± 3.7	0.019 ± 0.005	0.12 ± 0.03
C8	43.3 ± 5.5	0.117 ± 0.020	20.0 ± 3.4	0.025 ± 0.004	0.15 ± 0.03
C9	23.8 ± 7.0	0.064 ± 0.019	10.7 ± 3.1	0.013 ± 0.004	0.09 ± 0.02

The H_ex_ index allows for the assessment of the potential radiation hazard resulting from external exposure to gamma radiation emitted by isotopes naturally present in hard coal. This dimensionless quantity must be less than one to consider the ionising radiation emitted by a hard coal sample to be insignificant from the point of view of radiological protection (European Commission, 1999). The average calculated values of the radioactivity concentration index for coal samples are within the range of 0.052–0.156. The H_ex_ index values are significantly < 1 for the tested samples, indicating that the assessed external gamma radiation exposure from these materials remains well below the commonly applied screening threshold and is considered radiologically insignificant based on the indicators applied in this study.

The external worldwide average gamma radiation dose rate from natural isotopes present in the Earth’s crust is estimated at 54 nGy·h^−1^, assuming that the average contents of potassium, uranium (radium) and thorium in the Earth’s crust are 400 ± 24 Bq·kg^−1^, 37 ± 4 Bq·kg^−1^, 33 ± 3 Bq·kg^−1^ (UNSCEAR, 2008). The average and range of external gamma radiation dose rate in Poland from natural radionuclides of radium, thorium and potassium contained in the Earth’s crust is 47.4 nGy·h^−1^ and 18.8–86.0 nGy·h^−1^ (European Commission, 1999). The gamma radiation dose rate from the tested hard coals was in the range of 1.74–25.90 nGy·h^−1^. For none of the tested samples was an increase in the external gamma radiation dose rate in the environment observed, both in relation to the global average and the average for Poland. In none of the tested samples was a higher dose rate of external gamma radiation in the environment observed, both in relation to the worldwide average and the average for Poland. However, calculations based on literature data (Table 5) indicate that the gamma radiation dose rate from world hard coal deposits may be similar to Polish resources (from 6 nGy·h^−1^) or much higher, even up to 170 nGy·h^−1^.

**Table 5:** Values of radiological protection parameters for hard coals based on the literature

Sample ID	Ra_eq_ (Bq·kg^−1^**)**	H_ex_ (-)	D_ex_ (nGy·h^−1^**)**	AED_out_ (mSv·y^−1^**)**	I_γ_ (-)
C10	37	0.10	17	0.021	0.14
C11	46	0.12	21	0.026	0.16
C12	117	0.32	54	0.066	0.42
C13	83	0.22	37	0.046	0.29
C14	87	0.23	39	0.047	0.31
C15	119	0.32	55	0.068	0.42
C16	82	0.22	38	0.046	0.30
C17	86	0.23	38	0.047	0.30
C18	367	0.99	170	0.208	1.24
C19	97	0.26	43	0.053	0.34
C20	205	0.55	91	0.112	0.72
C21	48	0.13	21	0.026	0.17
C22	95	0.26	42	0.052	0.33
C23	20	0.05	9	0.011	0.07
C24	18	0.05	8	0.010	0.07
C25	188	0.51	80	0.098	0.66
C26	133	0.36	59	0.072	0.47
C27	68	0.18	30	0.037	0.24
C28	71	0.19	32	0.039	0.25
C29	50	0.13	22	0.028	0.18
C30	26	0.07	12	0.015	0.09
C31	36	0.10	17	0.021	0.13
C32	26	0.07	12	0.015	0.09
C33	49	0.13	23	0.028	0.16
C34	12	0.03	6	0.007	0.04
C35	289	0.78	134	0.165	1.03
C36	129	0.35	60	0.074	0.46
C37	1216	3.28	521	0.639	4.30
C38	247	0.67	114	0.139	0.86
C39	86	0.23	40	0.049	0.32
C40	62	0.17	28	0.034	0.22
C41	61	0.17	27	0.033	0.21
C42	51	0.14	23	0.028	0.18
C43	44	0.12	20	0.024	0.15
C44	34	0.09	15	0.018	0.12
C45	19	0.05	9	0.010	0.07
C46	34	0.09	15	0.018	0.12
C47	23	0.06	11	0.013	0.08

According to UNSCEAR (UNSCEAR, 2008), the average annual effective dose due to external exposure to natural terrestrial sources of radiation is 0.066 mSv·y^−1^ (Drzymała et al., 2020). For the coal samples tested, the average external annual effective dose ranged from 0.002 to 0.032 mSv·y^−1^. For none of the observed coal samples was the average external annual effective dose observed to exceed the values recommended by UNSCEAR (UNSCEAR, 2008) and ICRP, 2007 (ICRP, 2007), i.e., below one millisievert per year. The AED not exceed recommended values also for none coal sample described in analysed literature and presented in Table 5 (Akkurt et al., 2009; Cevik et al., 2008; Charro & Pena, 2013; Coles et al., 1978; Fardy et al., 1989; Flues et al., 2007; Habib et al., 2019; IAEA, 2003; Janković et al., 2011; Kolo et al., 2016; Lauer et al., 2015; Lu et al., 2006, 2012; Makundi et al., 2018; Mishra, 2004; Organo et al., 2004; Ozmen et al., 2024; Pak et al., 2021, 2025; Róg, 2005; Sahu et al., 2014; Smith et al., 2001). The determined radioactivity concentration index (Iγ) values for the samples ranged from 0.01 to 0.20. For all samples, the index values were below unity, meaning that the coals are radioactively safe and can be safely used in various industries. Additionally, the authors calculated the radiological indices Raeq, Hex, Dex, AEDout, and Iγ based on the concentrations of natural radioactive isotopes listed in Table 3 to verify whether the hard coals tested by other scientists are safe and do not cause an increase in the gamma radiation dose rate in the environment, and therefore do not expose humans to additional radiation doses (Table 5).

**Fig. 4:**
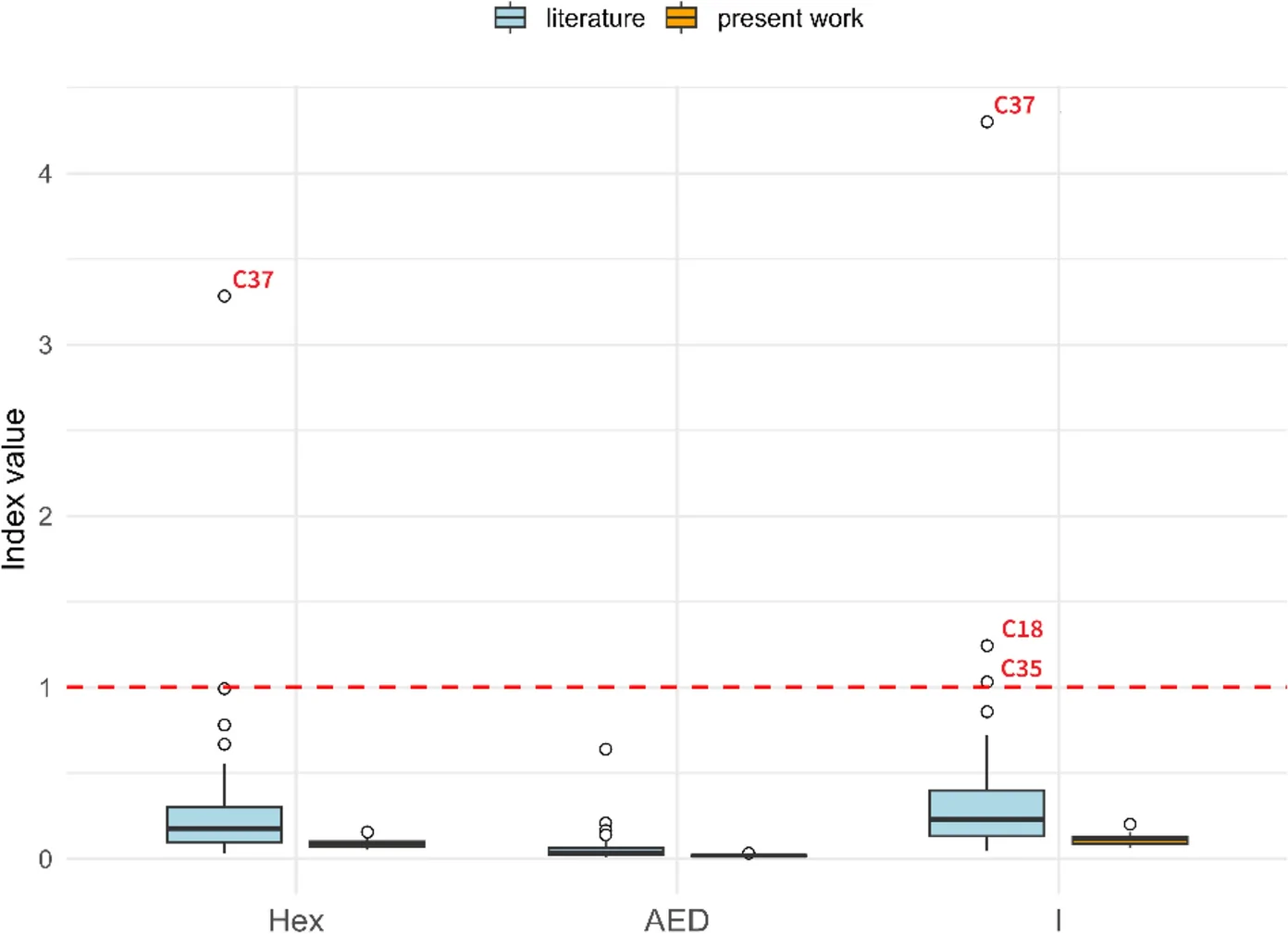
Boxplot comparison of the radiological hazard indices: external hazard index (H_ex_), annual effective dose (AED), and internal hazard index (Iγ) in coal samples from various countries, categorised by data source (present study in orange; literature in light blue). A red dashed line indicates the recommended threshold value of 1.0, as proposed by UNSCEAR. Only outlier points exceeding this threshold are annotated with sample identifiers

**Fig. 5:**
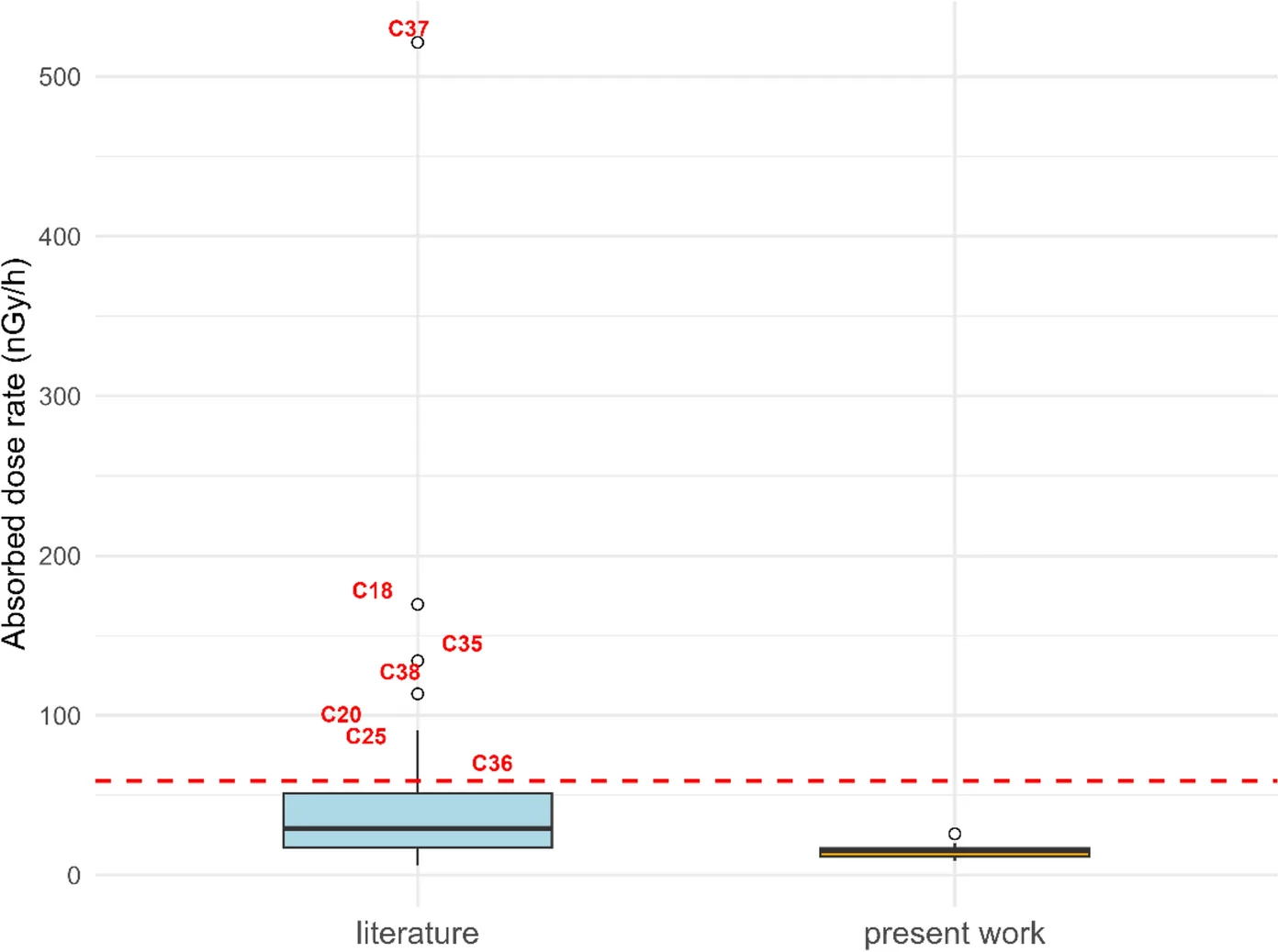
Boxplot of absorbed dose rate (D, in nGy·h^−1^) in coal samples from various countries, categorised by source of data (present study in orange; literature data in light blue). A red dashed line indicates the reference level of 59 nGy·h^−1^ recommended by UNSCEAR for outdoor exposure due to natural background radiation. Only samples with dose rates exceeding this value are labelled with their corresponding sample identifiers

**Fig. 6:**
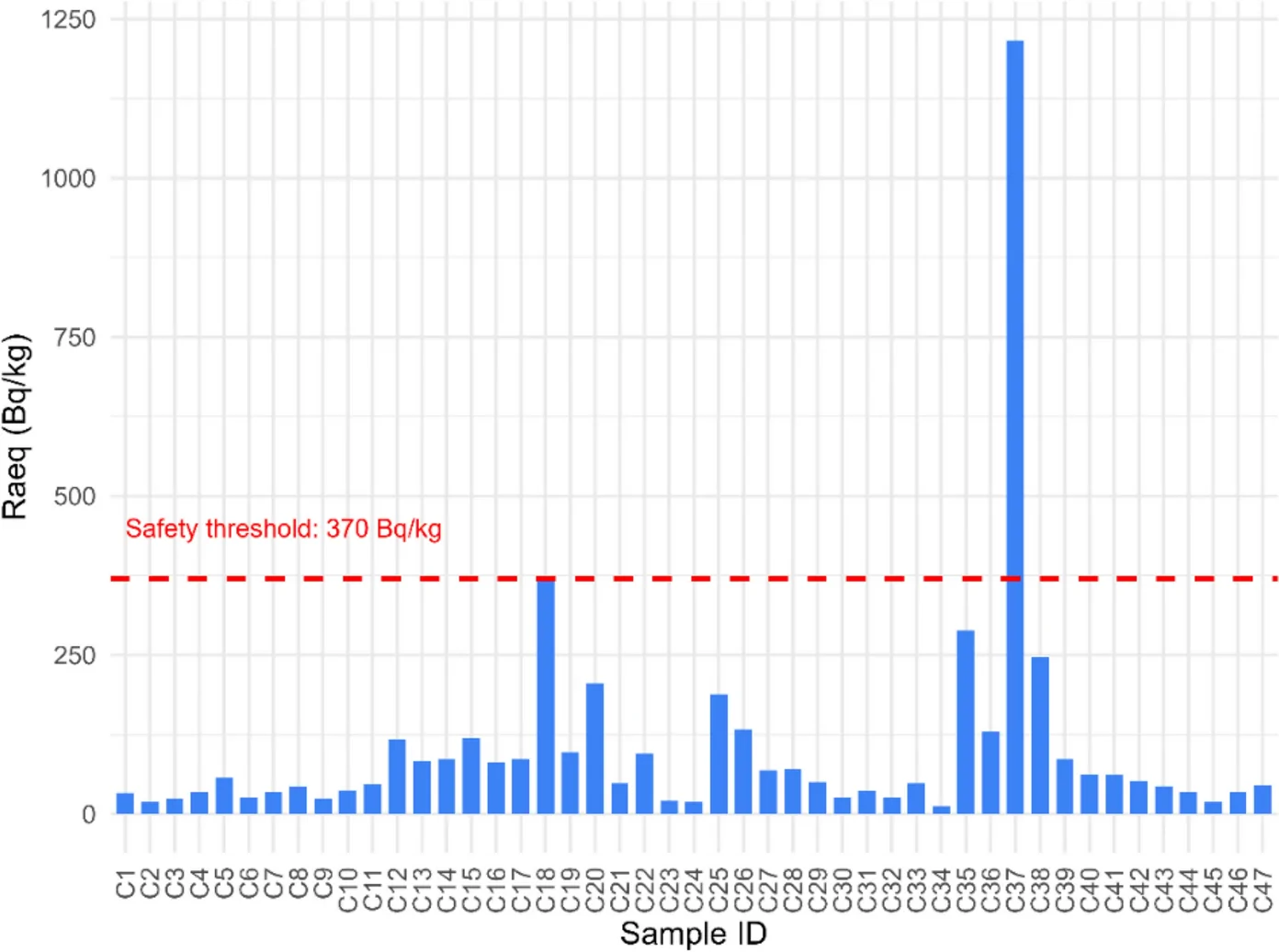
Bar plot of radium equivalent activity (Ra_eq_) for all analysed coal samples (C1–C47). The red dashed line indicates the recommended safety threshold of 370 Bq·kg^−1^, as defined by UNSCEAR guidelines

Figure 4 contains boxplots of the radiological hazard indices: external hazard index (H_ex_), annual effective dose (AED), and internal hazard index (Iγ) in coal samples from various countries, categorised by data source. Figure 5 contains boxplots of the absorbed dose rate values, while Fig. 6 shows radium equivalent values. Samples exceeding thresholds recommended by UNSCEAR may pose potential radiological risks and warrant further investigation.

The radiological indicator values calculated by the authors (Table 5) for hard coals from around the world indicate that not all samples are safe in terms of radiological protection. The radium equivalent concentration (Ra_eq_) and external radiation exposure index (H_ex_) values for the coal samples were within the ranges of 18–1216 Bq·kg^−1^ and 0.05–3.28, respectively. Increased or comparable values were obtained for samples from Russia (C37) and Brazil (C18). For the remaining coal samples, the global average Raeq and Hex values were not exceeded. The external gamma radiation dose rate (D_ex_) and annual external effective dose (AED_out_) values were within the ranges of 8–521 nGy·h^−1^ and 0.010–0.639 mSv·y^−1^, respectively. Elevated or comparable to the recommended D_ex_ and AED_out_ values were obtained for samples from Russia (C37), Brazil (C18), Germany (C35), Turkey (C38) and (C25), Bangladesh (C20), Ukraine (C36), Australia (C26), Turkey (C15), and Tanzania (C12). For the remaining samples, the external gamma radiation dose rate and external annual effective dose values were below their global averages. The radioactive concentration index values for coals ranged from 0.07 to 4.30. Elevated I_γ_ values were obtained for coal samples from Russia (C37), Brazil (C18), and Germany (C35). For the remaining coals, the I_γ_ values were significantly below unity. Considering the index values summarised in Table 5, it can be concluded that hard coals from Russia, Brazil, and Germany exhibit elevated values for all radiological indicators. Given the elevated radiological indicators, these materials may require increased radiological monitoring during their use and management to ensure compliance with applicable safety standards. Furthermore, burning hard coal with elevated concentrations of radium, thorium, and potassium can result in the production of highly radioactive combustion byproducts (fly ash, slag, ash-slag mixtures) due to the concentration of radioactive isotopes in these materials. This means that managing such waste in a circular economy can pose significant challenges. Currently, as an EU member, Poland promotes a circular economy in which waste is treated as a resource, not a problem. Therefore, it would be advisable to implement monitoring of natural radioactive isotope concentrations in imported hard coal.

These results are consistent with previous studies indicating that most hard coals do not pose a significant radiological hazard in their natural state. However, literature data (Table 5) demonstrate that certain coal deposits, particularly those from Russia (Pak et al., 2021), Brazil (Flues et al., 2007), and Germany (IAEA, 2003), may exhibit substantially elevated radiological parameters. This variability highlights the importance of considering geological origin when assessing the radiological safety of coal.

### Statistical analysis

*Normality assessment.* The Shapiro–Wilk test was applied to assess the normality of the activity concentration distributions of ^40^K, ^226^Ra, and ^232^Th across all coal samples (n = 47), combining the nine samples analysed in the present study with thirty-eight literature-derived samples compiled in Table 3. The test results yielded W statistics well below 1 and extremely low p-values for each radionuclide:

- ^40^ K: W = 0.6747,* p* = 1.73 × 10^−8^
- ^226^Ra: W = 0.4716,* p* = 1.20 × 10^−11^
- ^232^Th: W = 0.2780,* p* = 6.10 × 10^−13^

Given that all* p*-values are far below the conventional significance level (α = 0.05), the null hypothesis of normality was rejected for all three radionuclides. These results indicate that the distributions of ^40^K, ^226^Ra, and ^232^Th deviate significantly from the normal distribution. As a result, non-parametric methods are warranted for analysis. The reported uncertainties for the activity concentrations are the combined standard uncertainties, multiplied by a coverage factor of k = 2. This defines an expanded uncertainty corresponding to a confidence level of approximately 95% (2σ). The combined standard uncertainty includes components from counting statistics, detector efficiency calibration, and background measurement.

*Correlation analysis.* Spearman’s rank-order correlation was used to assess the monotonic relationships between the activity concentrations of the naturally occurring radionuclides ^40^K, ^226^Ra, and ^232^Th in coal samples. Given the non-normal distributions (as established by the Shapiro–Wilk test), this non-parametric method was chosen as the appropriate alternative to Pearson’s correlation. The analysis revealed statistically significant positive correlations across all radionuclide pairs. The correlation between ^40^K and ^226^Ra showed a moderate positive relationship (ρ = 0.54,* p* = 0.00019), indicating that samples with higher potassium activity also tended to exhibit elevated levels of radium. A slightly stronger association was observed between ^40^K and ^232^Th (ρ = 0.59,* p* = 0.00011), suggesting a notable co-distribution of potassium and thorium. The correlation between ^226^Ra and ^232^Th was also statistically significant and positive, though slightly weaker (ρ = 0.50,* p* = 0.00083). These findings imply that the radionuclides may be co-associated within similar mineral phases or originate from geochemically related sources in the coal matrix. The presence of monotonic relationships suggests consistent patterns of co-enrichment, potentially reflecting shared depositional or diagenetic processes. While the presence of tied ranks in the dataset precluded exact p-value computation, the approximate values remained robust and well below the conventional significance threshold (α = 0.05).

*Outliers detection.* The IQR (interquartile range) method was employed to identify potential outliers in the dataset for both natural radionuclides (^40^ K, ^226^Ra, and ^232^Th) and derived radiological hazard indices (Ra_eq_, H_ex_, D, AED, and I). Given that none of these variables followed a normal distribution, non-parametric techniques were appropriate for detecting extreme values. Table 6 contains the results of the IQR outliers analysis.

**Table 6:**
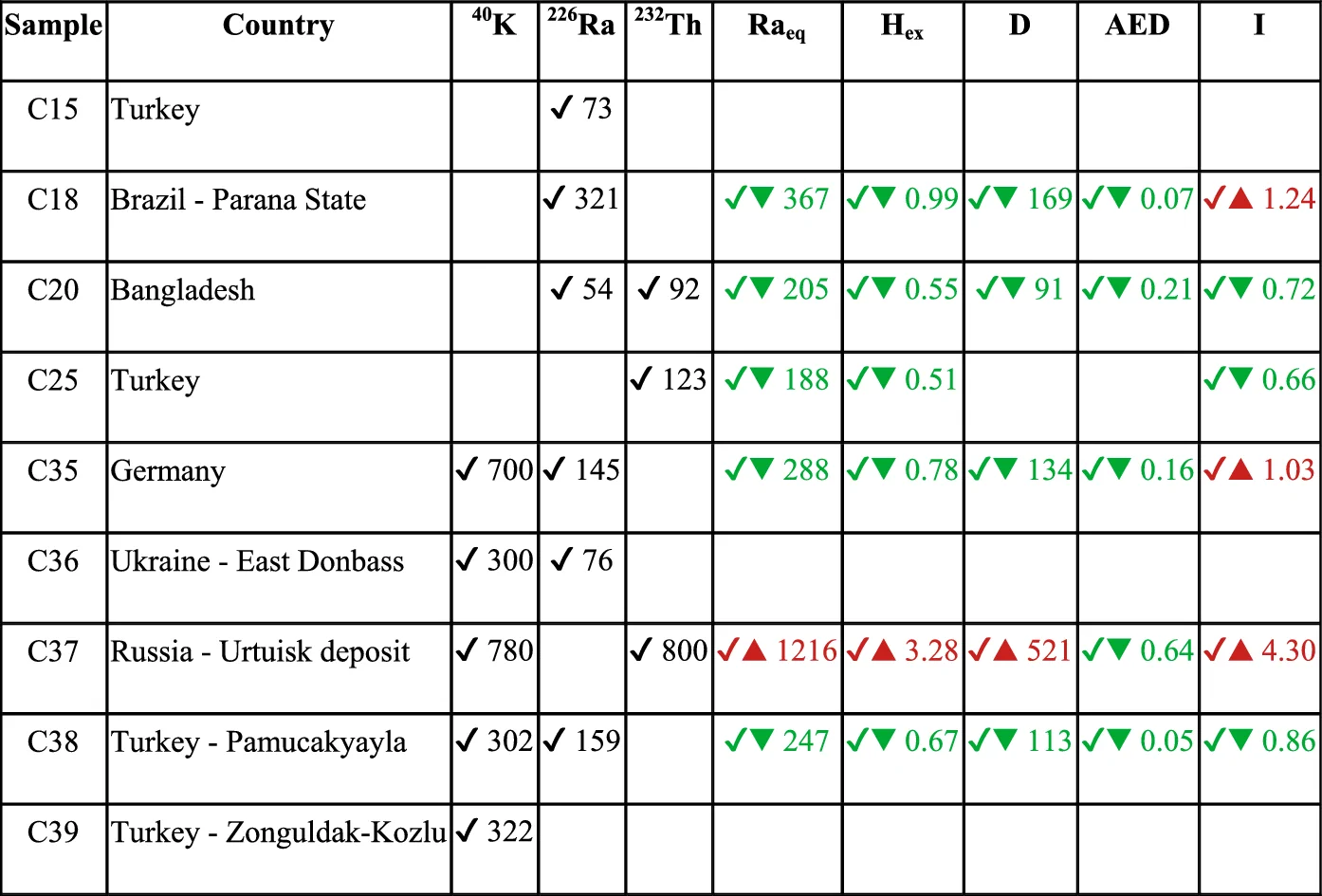
Summary of detected statistical outliers for radionuclide activity concentrations and radiological hazard indices

*Outliers for natural radionuclides are as follows:* ^40^K: Five outliers were identified, notably samples C35, C36, C37, C38, and C39, with potassium activity concentrations ranging from 300 to 780 Bq·kg^−1^. These values are significantly elevated relative to the typical sample range, with C37 and C35 being especially enriched. ^226^Ra: Six samples exceeded the upper threshold, including C18 (321 Bq·kg^−1^) and C38 (160 Bq/kg), suggesting notable radiological enrichment in those samples. ^232^Th: Three outliers were found, most prominently C37 (800 Bq·kg^−1^)—a value far above all others. Additional samples, such as C25 and C20, also presented elevated thorium levels.

*Outliers for radiological hazard indices:* Ra_eq_: Outlier samples included C37 (1216 Bq·kg^−1^) and C18 (367 Bq·kg^−1^), both substantially exceeding the 370 Bq·kg^−1^ safety reference level commonly cited in literature. Hex: Six outliers were detected, with C37 (H_ex_ = 3.28) far exceeding the radiological safety threshold of 1.0 recommended by UNSCEAR. This indicates that sample C37 poses a considerable external radiation hazard. D (absorbed dose rate): Five samples were identified as outliers, most notably C37 (521 nGy·h^−1^) and C18 (170 nGy·h^−1^), both of which far exceed the 59 nGy·h^−1^ reference level for outdoor exposure. AED (Annual Effective Dose): Sample C37 again stood out with 0.639 mSv·y^−1^, more than triple the commonly referenced threshold of 0.07 mSv·y^−1^ for global mean exposure from natural sources. I (Internal Hazard Index): Sample C37 was again the most extreme outlier (I = 4.30), followed by C18 and C35, all exceeding the UNSCEAR-recommended limit of 1.0.

Overall, sample C37 consistently emerged as an extreme outlier across all radionuclide concentrations and derived hazard indices, indicating a potentially anomalous or geochemically distinct coal source with elevated radiological risks. Other samples, such as C18, C35, and C38, also repeatedly appeared as outliers, though to a lesser extent.

*Comparison of the activity concentration of the radionuclides.* The Friedman rank sum test was performed to evaluate whether the median activity concentrations significantly differ among the three radionuclides: ^40^K, ^226^Ra, and ^232^Th, measured within the same set of samples. The test yielded a Friedman chi-squared statistic of 42.316 with 2 degrees of freedom. The associated p-value was 6.475 × 10^−10^. Given that the p-value is far below the conventional significance level (α = 0.05), we reject the null hypothesis, which stated that the distributions of activity concentrations for all three radionuclides are identical across the matched samples. There is strong statistical evidence that at least one of the radionuclides exhibits a significantly different distribution of activity concentration compared to the others within the same sample set.

To identify which specific pairs differ significantly, a post-hoc analysis was conducted: Nemenyi post-hoc test as well as pairwise Wilcoxon test with Bonferroni correction (Table 7).

**Table 7:** Pairwise statistical comparisons of radionuclide activity concentrations using Wilcoxon signed-rank and Nemenyi post-hoc tests

Comparison	Adjusted *p*-value	Significance	Nemenyi *p*-value	Nemenyi significance
40 K versus 226Ra	3.80E−06	***	4.30E−06	***
40 K versus 232Th	1.10E−07	***	1.80E−09	***
226Ra versus 232Th	3.60E−01	n.s	3.50E−01	n.s

Significance levels are indicated by asterisks: * *p* < 0.05, ** *p* < 0.01, *** *p* < 0.001. The label n.s. denotes non-significant differences (*p* ≥ 0.05)

The Nemenyi-Wilcoxon-Wilcox all-pairs test comparisons revealed the following: there is a highly significant difference between ^40^K and ^226^Ra (*p* = 4.3 × 10^−6^). There is also a highly significant difference between ^40^K and ^232^Th (*p* = 1.8 × 10^−9^), however, the difference between ^226^Ra and ^232^Th was not statistically significant (*p* = 0.35). These findings indicate that the activity concentration of ^40^K differs significantly from both ^226^Ra and ^232^Th, while no significant difference was detected between the latter two radionuclides. This pattern suggests that potassium-40 exhibits a distinct radiological behaviour or distribution in the analysed coal samples, compared to thorium and radium isotopes.

The adjusted* p*-values for the pairwise comparisons in the Wilcoxon signed-rank test are as follows: ^40^K vs ^226^Ra:* p* = 3.8 × 10^−6^—highly significant; ^40^K vs ^232^Th:* p* = 1.1 × 10^−7^—highly significant; ^226^Ra vs ^232^Th:* p* = 0.36—not statistically significant. These findings confirm that the activity concentration of ^40^K is significantly different from both ^22^⁶Ra and ^232^Th, whereas the concentrations of ^226^Ra and ^232^Th do not differ significantly from one another in the same sample set. This pattern mirrors the results obtained from the Nemenyi post-hoc analysis, thereby reinforcing the robustness of the observed differences involving potassium ^40^ K.

### The effect of hard coal combustion on the concentration of natural radioactive isotopes in the bottom ash

Ash in coal is defined as a non-combustible mineral component of the raw material that remains after the coal combustion process. The ash content of coal can vary significantly, ranging from several to several dozen percent of its mass, depending on the specific type of coal. The ash content in coal is one of the basic parameters determining coal quality and its utilisation. The radiological assessment of ash is of crucial importance due to the fact that large quantities of ash from the energy sector are utilised for a variety of purposes, most commonly in road and building construction, and occasionally, in combination with sewage sludge, as a fertiliser in agriculture. Consequently, elevated radionuclide concentrations can exert considerable influence on the environment and human health. In the present study, bottom ash was selected for analysis rather than fly ash, as it constitutes the fraction directly collected from the combustion chamber of the domestic grate-fired boiler used in the experiment—fly ash was not separated in this combustion setup. It should be acknowledged, however, that fly ash generated in industrial power plants typically exhibits even higher radionuclide concentrations than bottom ash, due to preferential association of fine-grained mineral phases with the lighter particle fraction (Ahmed & Joubert, 2019; Celenlioglu & Karali, 2025). The enrichment factors reported in this study may therefore be considered conservative relative to those expected for fly ash fractions in industrial-scale combustion systems. The elevated radionuclide concentrations observed in combustion residues result primarily from a mass balance effect: during combustion, the organic fraction of coal is oxidised and removed as gaseous products, while mineral-associated radionuclides are retained in the solid residue. As the total mass of the material is substantially reduced, the same absolute quantity of radionuclides becomes concentrated in a smaller mass, leading to a proportional increase in activity concentration per unit mass. Although ash yield was not directly gravimetrically determined for individual samples, it was estimated from the inverse of the ^22^⁶Ra enrichment factor—a well-established indirect approach. The estimated ash yields ranged from approximately 10% to 27% of the original coal mass, with a median of ~20%, consistent with typical values reported for bituminous coals in the literature. These estimates confirm that the observed radionuclide enrichment is primarily governed by the reduction in material mass following combustion of the organic fraction. Similar observations were made in other studies (Bhangare et al., 2014; Fidanchevski et al., 2021; Jalil & Rashid, 2015).

Previous studies have shown that combustion of hard coal typically results in enrichment of natural radionuclides in ash by a factor of approximately 2–5, depending on coal composition, combustion technology, and ash fraction analysed. For instance, Mora et al. (2009) reported enrichment factors of 2–3 in bottom ash from Spanish coal-fired power plants, while Bhangare et al. (2014) and Sahu et al. (2014) observed 3–5-fold increases for various radionuclides in Indian thermal power stations. Comparable enrichment ranges were also reported for coal combustion residues in Malaysia and Serbia.

In contrast, the present study demonstrates a consistently higher amplification, with median ash-to-coal ratios clustering around ~6 for all investigated radionuclides and derived radiological indices. This systematic shift towards higher enrichment, combined with complete directional consistency across all coal-ash pairs, suggests that the observed amplification cannot be attributed solely to random variability or isolated sample characteristics.

The stronger and more uniform amplification observed in this study is likely related to the experimental design and material pairing employed. Unlike most literature studies, which assess coal and ash as independent sample sets originating from industrial-scale combustion systems, the paired coal–ash approach applied here directly links each ash sample to its parent coal. This excludes confounding factors related to fuel blending, variable residence times, and heterogeneous ash streams, which may partially obscure systematic enrichment patterns in large power plants.

Moreover, the combustion process applied in this study resulted in substantial removal of organic matter while preserving mineral-bound radionuclides, leading to concentration in a reduced mass fraction. The convergence of ash-to-coal ratios across independent radiological parameters further indicates that the amplification is governed primarily by mass balance effects rather than radionuclide-specific geochemical behaviour.

In case of present study, the bottom ash, being a by-product of combustion, is characterized by a significantly elevated concentration of natural radioactive isotopes in comparison to the coals from which the ashes originated (Table 2 and 8). The enrichment factors (EF) of all investigated radionuclides exhibit values between 4 and 10. The lowest values of enrichment factors (up to 5.37) are indicative of ^226^Ra, with the exception of coal from Colombia, which has an EF ^226^Ra equal to 9.89. Increase in ^232^Th and ^40^K in ash was higher: EF was in the range between 5 and 10. The most significant enrichment of all radionuclide concentrations in ash is observed for samples from Colombia, the USA, and Kazakhstan.

**Table 8:** Concentration of natural radioactive isotopes ^226^Ra, ^232^Th and ^40^ K in the bottom ash (BA) samples and the enrichment factors (in brackets) in relation to coal samples

Origin country	Sample	^226^Ra (Bq·kg^−1^)	^232^Th (Bq·kg^−1^)	^40^ K (Bq·kg^−1^)
Australia (Hail Creek)	BA1	83.0 ± 7.0 (5.37)	55 ± 4 (5.00)	124 ± 11 (5.10)
Poland (Poland Mix)	BA2	78.0 ± 6.0 (5.04)	48 ± 3 (-)	−
Kazakhstan	BA3	85.0 ± 7.0 (4.99)	36 ± 4 (10.00)	167± 9 (7.05)
USA (Virginia)	BA4	104.0 ± 9.0 (5.03)	64 ± 3 (7.62)	161 ± 10 (5.95)
Mozambique	BA5	125 ± 9.0 (4.94)	187 ± 9 (9.49)	330 ± 17 (5.98)
Australia PLV	BA6	61 ± 6 (4.78)	81 ± 6 (9.31)	117 ± 10 (-)
USA (Clintwood)	BA7	112 ± 8 (4.96)	51 ± 3 (9.11)	423 ± 25 (9.34)
Poland USC	BA8	72 ± 7 (3.75)	61 ± 8 (5.30)	401 ± 23 (4.03)
Colombia	BA9	56 ± 6 (9.89)	92 ± 8 (9.20)	259 ± 24 (7.18)

The observed enrichment is consistent with the well-established mechanism of radionuclide concentration during combustion, whereby the organic fraction of coal is oxidised and removed, while mineral-associated radionuclides remain in the solid residue. As a result, radionuclides become concentrated in a reduced mass of material. Similar enrichment effects have been reported in previous studies, typically within a range of 2–5 times higher activity in ash compared to coal. However, the enrichment factors observed in this study, reaching values up to approximately 10 and clustering around a median of ~6, indicate a more pronounced and consistent amplification effect.

To determine whether the observed enrichment represents a systematic effect of combustion rather than isolated sample-specific behaviour, a paired coal-ash analysis was performed. As shown in Table 9, the transition from coal to bottom ash resulted in a highly consistent increase in radionuclide activity concentrations and radiological hazard indices across all analysed samples.

**Table 9:** Paired coal-ash comparison of radionuclide activity concentrations and radiological hazard indices: median values, ash-to-coal ratios, and effect sizes

Parameter	Median (Coal)	Median (Ash)	Median A/C ratio	Min–Max A/C	*p*-value*	Effect size (r)
^40^K (Bq·kg⁻^1^)	27.0	259	5.98	4.03–65.1	0.0038	0.889
^226^Ra (Bq·kg⁻^1^)	17.0	83	4.99	3.75–9.89	0.0038	0.889
^232^Th (Bq·kg⁻^1^)	8.7	61	9.20	5.00–20.9	0.0038	0.889
Raeq (Bq·kg⁻^1^)	33.1	190	6.40	4.38–9.42	0.0038	0.889
Hex (-)	0.089	0.51	6.41	4.36–9.39	0.0038	0.889
D (nGy·h⁻^1^)	14.8	87	6.37	4.35–9.51	0.0038	0.889
AED (mSv·y⁻^1^)	0.018	0.103	6.14	1.38–9.51	0.0038	0.889
Iγ (-)	0.115	0.68	6.51	4.39–9.78	0.0038	0.889

It is important to note that the combustion of any type of coal can result in the emission of radioactive isotopes, thereby posing a potential threat to the environment. In order to assess a possible health effect caused by exposure of humans to natural radionuclides present in the ashes, the radiological hazard indices: the radium equivalent activity (Ra_eq_), external concentration index (H_ex_), external gamma radiation dose rate (D_out_), external annual effective dose (AED_out_), and radioactivity concentration index (I_γ_) were calculated and compared to those of hard coal samples.

A/C denotes the ratio of the parameter value measured in bottom ash to that measured in the corresponding parent coal (A/C = ash/coal). Statistical significance was assessed using a paired Wilcoxon signed-rank test (alternative hypothesis: ash > coal; n = 9). Directional consistency indicates the number of coal-ash pairs for which the parameter value in ash exceeded that in coal.

**Fig. 7:**
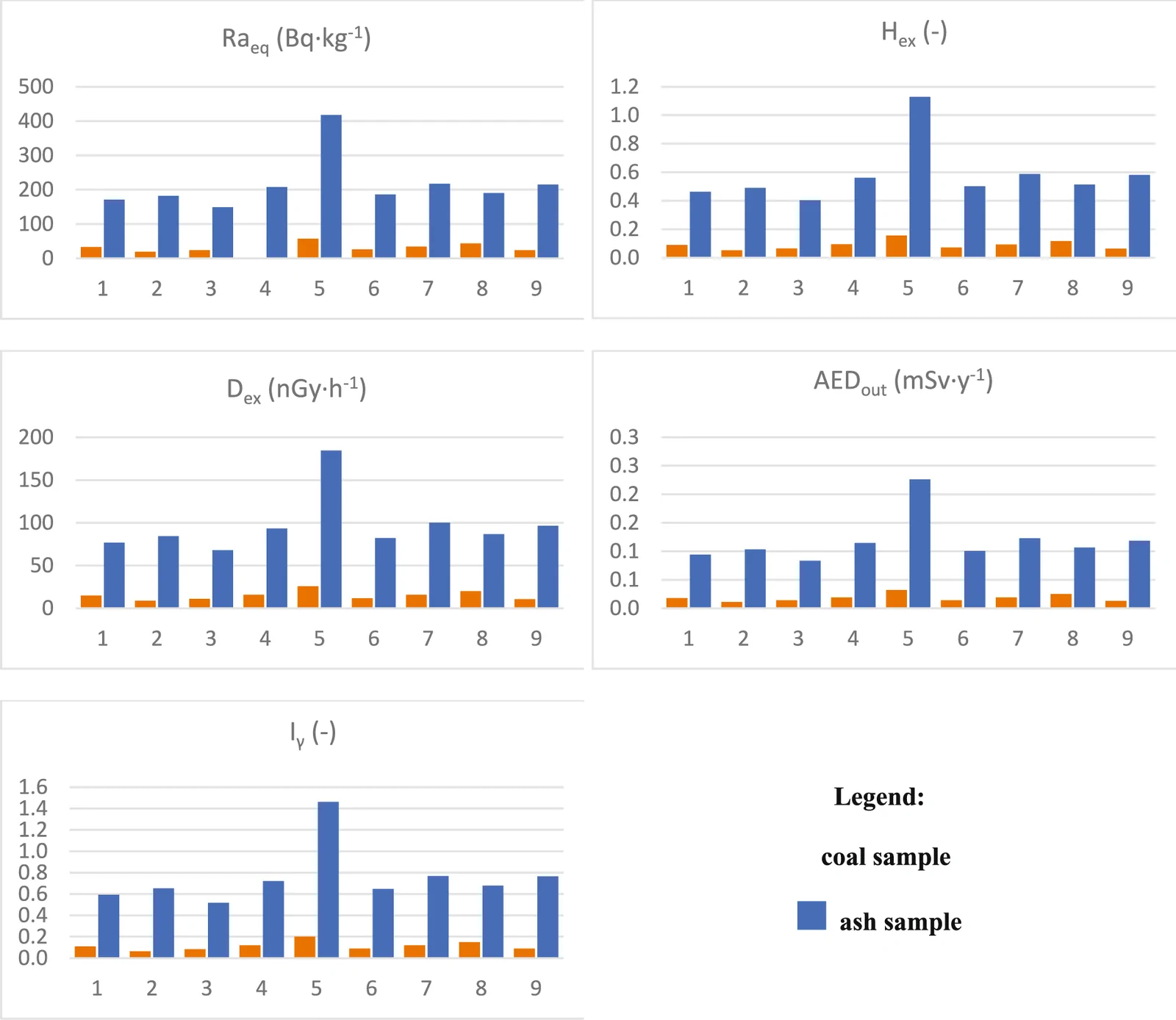
Bar plot of the radiological hazard indices: the radium equivalent activity (Ra_eq_), external concentration index (H_ex_), external gamma radiation dose rate (D_ex_), external annual effective dose (AED_out_), and radioactivity concentration index (I_γ_) of all analysed coal and ash samples–for comparison

Unlike most previous studies, which analyse coal and ash independently, the paired coal–ash approach applied here enables direct quantification of combustion-induced transformation. The high directional consistency and very large effect sizes observed across all parameters demonstrate that this process is not random, but systematic and reproducible. This suggests that combustion acts as a proportional amplifier of natural radioactivity, allowing the radiological properties of ash to be inferred from those of the parent coal. As these indices strictly depend on the concentration of radioactive isotopes in the sample, their values for ash are considerably elevated in comparison to those for coals, and their range is as follows:

Ra_eq_: 149 − 418 Bq·kg^−1^; H_ex_: 0.46 − 1.13; D_out_: 68 − 185 nGy·h^−1^; AED_ou_: 0.018 − 0.226 mSv·y^−1^; I_γ_: 0.52 − 1.46. The median values of these indices are similar to the results obtained for bottom ash from thermal power plants in the Republic of North Macedonia (Fidanchevski et al., 2021). The highest values were recorded for sample BA5, which originates from coal seams in Mozambique. As illustrated in Fig. 7, samples BA3 and BA1, followed by BA6 exhibited the lowest radiological parameter values. Contrary to the elevated values of these indices for ashes in comparison to coal, the majority of the samples (with the exception of BA5) do not surpass the acceptable limits of Ra_eq_, H_ex_, AED_out_ and I_γ_, determined by UNSCEAR and ICRP guidelines. It should be noted, however, that values of the external gamma radiation dose rate (D_ex_) for all ash samples exceed the recommended reference value of 54 nGy h⁻^1^ (Jalil & Rashid, 2015; Pandit et al., 2011). This refers specifically to the absorbed gamma dose rate, whereas the remaining radiological indicators (Ra_eq_, H_ex_, AED_out_, and Iγ) remain below their respective reference levels in the majority of cases, indicating that the overall radiological risk profile of the analysed ashes is not uniformly elevated across all assessed parameters.

In the case of samples from Poland and Australia, reliable measurement of radioactivity was difficult due to the minimal amount of ash obtained during the combustion process. Ash yield is a recognised indicator of coal quality, as it is inversely related to calorific value and directly influences the commercial grading and industrial usage of coal (Mishra et al., 2025). Accordingly, hard coal from Poland and Australia analysed in this study appears, in this respect, to constitute a comparatively higher-quality fuel relative to the other coal types examined.

The paired coal-ash analysis revealed a highly consistent and quantitatively robust radiological transformation induced by combustion. For all analysed radionuclides and derived radiological hazard indices, the ash-to-coal ratios clustered tightly around a value of approximately six, irrespective of the physical quantity considered. This convergence across independent indicators demonstrates that combustion acts as a proportional radiological amplifier rather than producing isolated or radionuclide-specific effects.

Despite the limited number of samples, the magnitude of the observed changes was substantial, as reflected by very large effect sizes (r = 0.889 for all parameters) and complete directional consistency across all coal-ash pairs. Such results indicate that the enrichment of natural radionuclides and associated radiological indices in bottom ash is a predictable consequence of organic matter removal and mineral phase retention during combustion. Other researchers also observed a 2–5 times higher radioactivity of ash than coal (Bhangare et al., 2014; Mora et al., 2009). A significant enrichment effect of radionuclides in bottom ash was also observed in studies of coals burned at a power plant in Spain (Mora et al., 2009) and Malaysia (Jalil & Rashid, 2015). The present study extends this understanding by demonstrating that the amplification is not only present, but also quantitatively consistent across multiple parameters.

When combined with the literature data presented in Sect. “Assessment of radiological indicators”, the results suggest that combustion of coals characterised by elevated radionuclide concentrations may produce ash that exceeds multiple radiological reference levels, posing challenges for waste management and circular economy applications (Pandit et al., 2011).

From an environmental perspective, the distinction between a 2–3-fold and a ~6-fold enrichment is not trivial. When coal combustion residues are reused at large scale in construction materials, road subbases, or land reclamation, even moderate differences in enrichment factors can translate into substantially different exposure scenarios. In this context, the paired approach presented here suggests that radiological risk assessments based solely on average ash concentrations reported in literature may underestimate potential impacts if coal-specific transformation factors are not considered.

### Environmental implications

The environmental relevance of these findings becomes particularly significant when considered in light of the approximately six-fold increase in radionuclide activity and radiological indices demonstrated in Sect. “The effect of hard coal combustion on the concentration of natural radioactive isotopes in the bottom ash”, combined with the scale of coal combustion and the widespread reuse of its by-products. The observed systematic enrichment of radionuclides in bottom ash has important environmental implications, particularly in the context of large-scale generation and reuse of coal combustion residues. Coal ash is widely utilised in construction materials, road base layers, and land applications, which creates potential pathways for the dispersion of naturally occurring radioactive materials into soil, groundwater, and the atmosphere. In such scenarios, even moderate initial radionuclide concentrations in coal may lead to environmentally relevant levels in secondary materials following combustion.

The approximately six-fold increase in radionuclide activity and radiological indices observed in this study indicates that combustion acts as a consistent and predictable amplification process. This finding is particularly relevant for environmental risk assessment, as it demonstrates that the radiological characteristics of coal cannot be directly extrapolated to its combustion residues without accounting for transformation processes. As a result, materials considered radiologically safe in their original form may contribute to increased environmental exposure after combustion.

From a regulatory and waste management perspective, these results highlight the need to treat coal combustion residues as radiologically distinct materials, especially in circular economy systems where such by-products are intentionally reintroduced into the environment. Incorporating process-based transformation factors into environmental assessment frameworks may improve the accuracy of risk evaluation and support the development of more effective monitoring and management strategies for TENORM-containing materials. In the European Union context, the management of NORM and TENORM materials is governed by the Euratom Basic Safety Standards (Council Directive 2013/59/Euratom), which establish activity concentration reference levels for building materials (e.g., an activity concentration index I ≤ 1 corresponding to an annual effective dose of 1 mSv) and require member states to identify and regulate industrial activities producing significant quantities of NORM-containing residues. The results of the present study—particularly the systematic exceedance of the Dex reference level and the elevated Iγ values observed for the Mozambique ash sample—indicate that coal combustion residues may fall within the scope of these regulations when used in construction applications, underscoring the practical relevance of process-based radiological transformation assessment.

## Conclusions

Addressing the first objective of this study, the analysed hard coal samples, both domestic and imported and representing geologically diverse origins, exhibited low activity concentrations of natural radionuclides and associated radiological indices below global average values, indicating negligible radiological risk in their original form. However, the results clearly demonstrate that coal combustion induces a systematic and substantial transformation of radiological properties.

In line with the second objective, the paired coal-ash assessment revealed a consistent and approximately six-fold increase in radionuclide activity concentrations and all radiological hazard indices in bottom ash, with complete directional consistency and very large effect sizes. This indicates that combustion acts as a predictable amplifier of natural radioactivity under the investigated combustion conditions, rather than a stochastic process.

With respect to the third objective, although most ash samples remained within recommended radiological limits, the magnitude and consistency of enrichment highlight the potential for secondary environmental contamination, particularly in scenarios involving large-scale generation and reuse of coal combustion residues. Notably, the external gamma radiation dose rate (Dex) exceeded the recommended reference level of 54 nGy·h⁻^1^ in all analysed ash samples, and radiological indices for the Mozambique sample exceeded regulatory thresholds across multiple parameters. These findings emphasise that radiological assessments based solely on coal properties may underestimate the environmental impact of its combustion products.

Given the variability of radionuclide concentrations in coals from different geological origins, continuous monitoring of both domestic and imported coal is warranted. Incorporating process-based transformation effects into environmental risk assessment frameworks may improve the management of TENORM-containing materials and support safer circular economy practices. It should be noted that the presented conclusions refer to the specific set of coal samples and combustion conditions applied in this study and should not be automatically generalised to all coal types and combustion technologies.

Several limitations of this study should be acknowledged. The analysis was based on nine coal–ash pairs, which, while sufficient for the applied non-parametric statistical framework, limits the generalisability of the quantitative findings to other coal types and combustion contexts. Although the samples were collected using a representative sampling procedure from shipments of diverse geographical origins, they cannot be considered fully representative of the global variability of hard coal composition. Also, the combustion experiments were conducted using a domestic grate-fired boiler operating under natural draught conditions, which differ from industrial pulverised-coal or fluidised-bed combustion systems in terms of temperature profiles, residence times, and ash fractionation. The enrichment factors reported here should therefore be interpreted as indicative of the direction and approximate magnitude of radiological transformation during combustion, rather than as precise quantitative predictions applicable to all combustion technologies. Additionally, radionuclide concentrations were determined from repeated analytical measurements of a single prepared sample per coal–ash pair, rather than from independently prepared subsamples; future studies should incorporate independent subsampling to separate analytical repeatability from within-material compositional heterogeneity.

## Data Availability

The datasets generated and analysed during the current study are available from the corresponding author upon reasonable request.
